# Multiple-Resampling Cross-Spectral Analysis: An Unbiased Tool for Estimating Fractal Connectivity With an Application to Neurophysiological Signals

**DOI:** 10.3389/fphys.2022.817239

**Published:** 2022-03-07

**Authors:** Frigyes Samuel Racz, Akos Czoch, Zalan Kaposzta, Orestis Stylianou, Peter Mukli, Andras Eke

**Affiliations:** ^1^Department of Physiology, Faculty of Medicine, Semmelweis University, Budapest, Hungary; ^2^Department of Neurology, Dell Medical School, The University of Texas at Austin, Austin, TX, United States; ^3^Institute of Translational Medicine, Semmelweis University, Budapest, Hungary; ^4^Oklahoma Center for Geroscience and Healthy Brain Aging, Department of Biochemistry & Molecular Biology, The University of Oklahoma Health Sciences Center, Oklahoma City, OK, United States; ^5^Department of Radiology & Biomedical Imaging, School of Medicine, Yale University, New Haven, CT, United States

**Keywords:** fractal connectivity, scale-free, bivariate, multiple-resampling, spectral analysis, electroencephalography, MRCSA

## Abstract

Investigating scale-free (i.e., fractal) functional connectivity in the brain has recently attracted increasing attention. Although numerous methods have been developed to assess the fractal nature of functional coupling, these typically ignore that neurophysiological signals are assemblies of broadband, arrhythmic activities as well as oscillatory activities at characteristic frequencies such as the alpha waves. While contribution of such rhythmic components may bias estimates of fractal connectivity, they are also likely to represent neural activity and coupling emerging from distinct mechanisms. Irregular-resampling auto-spectral analysis (IRASA) was recently introduced as a tool to separate fractal and oscillatory components in the power spectrum of neurophysiological signals by statistically summarizing the power spectra obtained when resampling the original signal by several non-integer factors. Here we introduce multiple-resampling cross-spectral analysis (MRCSA) as an extension of IRASA from the univariate to the bivariate case, namely, to separate the fractal component of the cross-spectrum between two simultaneously recorded neural signals by applying the same principle. MRCSA does not only provide a theoretically unbiased estimate of the fractal cross-spectrum (and thus its spectral exponent) but also allows for computing the proportion of scale-free coupling between brain regions. As a demonstration, we apply MRCSA to human electroencephalographic recordings obtained in a word generation paradigm. We show that the cross-spectral exponent as well as the proportion of fractal coupling increases almost uniformly over the cortex during the rest-task transition, likely reflecting neural desynchronization. Our results indicate that MRCSA can be a valuable tool for scale-free connectivity studies in characterizing various cognitive states, while it also can be generalized to other applications outside the field of neuroscience.

## Introduction

Many dynamical systems ranging from functional brain networks (Achard et al., 2008; Ciuciu et al., 2014) through geophysical systems (Campillo and Paul, 2003; Marinho et al., 2013), natural phenomena (Mandelbrot, 1983) or meteorological data (Vassoler and Zebende, 2012) to financial markets (Podobnik and Stanley, 2008; He and Chen, 2011) have been shown to express scale-free (or *fractal*) correlations both in the univariate dynamics of their individual constituents, as well as in their interactions. In the former case the autocorrelation function of the process exhibits a slow decay, while in the latter case the same holds for the cross-correlation function of the two processes at hand. However, a key characteristic of both scenarios is that a power-law relationship can be established between the correlation and the scale of observation (Podobnik et al., 2008). Equivalently, the same phenomenon can also be assessed in the frequency domain, where the long-range coupling manifests as the power-law dependency of auto- or cross-spectral power (or density) on the frequency (Kristoufek, 2014). The power-law relationship is commonly characterized in the obtained fractal scaling exponent, which is referred to as the Hurst exponent (*H*) or the spectral slope (β) in the time and frequency domains, respectively, with an explicit equivalence between the two (Eke et al., 2000; Kristoufek, 2014). Given that identifying such long-term couplings between various brain regions can reveal novel implications on the functional organization of the brain – that cannot be identified otherwise via single-scale or scale dependent analyses –, fractal connectivity studies gained growing interest recently (Achard et al., 2008; Ciuciu et al., 2014; Stylianou et al., 2020, 2021; La Rocca et al., 2021).

There is a plethora of available methods for assessing coupling between dynamic systems, such as random matrix theory (Plerou et al., 1999), cross sample entropy (Richman and Moorman, 2000), or various techniques of non-linear time series analysis (Schreiber, 1999, 2000; Kantz and Schreiber, 2004). Accordingly, in line with the fact that identifying fractal coupling might be of interest for many disciplines besides neuroscience, numerous methods have been developed to assess such interactions both in the frequency and time domains (Kristoufek, 2017). Nevertheless, most of these methods disregard the fact that the empirical data under consideration might not only be composed of broadband fractal, but narrow-band oscillatory components as well. To assess the extent of the latter, if present, is not only important for a characterization of the bias it introduces in the estimation of fractal measures, but more importantly the two (i.e., fractal and oscillatory) signal components are also likely to reflect distinct underlying processes. This notion becomes exceedingly relevant in case of neurophysiological fluctuations, which are well known to appear as a composite of broad- and narrow-band activities in electrophysiological recordings (Gonzalez et al., 1999; He et al., 2010; He, 2014) and are often hypothesized to be products of vastly different generating mechanisms (Buzsaki and Draguhn, 2004; Buzsaki et al., 2012). The same notion also applies for functional connectivity of the brain, where, e.g., synchronized alpha activity might appear as a peak superimposed on the otherwise broadband cross coherence spectrum (Murias et al., 2007b). Consequently, if one is to assess the fractal properties of neural activity, either in the univariate or multivariate case, separation of the fractal component of the signal from the rest seems essential.

In case of univariate fractal analysis of electrocorticography (ECoG) recordings, He et al. (2010) utilized coarse-graining spectral analysis (CGSA) – a method first introduced and then improved upon by Yamamoto and Hughson (1991, 1993) – to trim the power spectra of the recorded signals from oscillatory peaks and thus render the estimation of spectral slopes unbiased. Briefly, CGSA exploits the self-affine property of fractal processes, namely that the statistical distribution of the data remains unaffected when the process is resampled at a different time scale (Mandelbrot and Van Ness, 1968). Practically, a fractal process will have the same power spectrum adjusted by the resampling factor after resampling, while in a harmonic signal the oscillatory peak gets relocated. In other words, for a given frequency this means that following resampling, power will remain non-zero if the process is fractal, while reduce to practically zero if the signal is simply periodic with the given frequency. Therefore, one can reconstruct the fractal power spectrum by computing the cross-spectrum of the original signal and its resampled version (Yamamoto and Hughson, 1991, 1993). Recently, an improved algorithm termed irregular resampling auto-spectral analysis (IRASA) was proposed by Wen and Liu (2016) for separating the fractal and oscillatory components of neurophysiological signals. IRASA builds on the same principle as CGSA, however, it eliminates many of its shortcomings – such as its inability to handle multiple oscillatory components that are interrelated via the scaling factor – by utilizing not only two but a set of non-integer rescaling factors. Specifically, while in CGSA time series are resampled with *h=2* and *h* = 1/2, IRASA uses a set of positive non-integer numbers between 1 and 2 and their reciprocals (e.g., *h* = 1.1 and *h* = 1/1.1 = 0.9091), hence the term ‘irregular-resampling’; even though the resampled time series – similarly to the original – are evenly (regularly) sampled.

Nevertheless, both CGSA and IRASA can only be utilized in the analysis of univariate signals (i.e., individual recordings). On the other hand, one can expect to face similar problems when investigating functional connectivity in the frequency domain: the broadband cross-coherency spectrum indicating fractal connectivity might well be interspersed with oscillatory peaks reflecting, e.g., the effect of large-scale cortical alpha synchronization even in the resting state (Murias et al., 2007b) or during cognitive stimulation (Murias et al., 2007a). For a better assessment and understanding of fractal connectivity, therefore, methods are called for that can eliminate the effects of such scale-dependent interactions and separate the scale-free component of statistical interdependence. This is the primary focus of this paper; here, we propose an extension of IRASA to the bivariate case, which we title multiple-resampling cross-spectral analysis (MRCSA) for isolating the fractal component of the cross-spectral density of a pair of neurophysiological signals. We show that MRCSA can yield a theoretically unbiased estimate of the fractal cross-spectrum and thus the cross-spectral slope, and although it is unable to purely separate oscillatory components due to the potential presence of complex interactions between the fractal and oscillatory components, it can provide useful information on the contribution of fractal connectivity via assessing the proportion of fractal to total cross-spectral power. We utilize simulations to present the robustness of MRCSA against the presence of a high number of synchronized oscillatory components. Finally, we demonstrate the real-world applicability of MRCSA on experimental electroencephalography (EEG) recordings by analyzing fractal connectivity in two different conditions, resting state and word generation.

## Materials and Methods

First, we introduce the self-affine property of fractal processes in contrast to oscillatory signals. Then, we briefly summarize the CGSA and IRASA methods to show how the fractal component of the power spectrum can be separated in the univariate case. Finally, we show that the IRASA pipeline can be extended to the bivariate case, where it provides an unbiased estimate on the fractal component of the cross-spectrum. In what follows we describe the method used for simulating time series with known cross-spectral slope and oscillatory peaks and use the simulated time series to demonstrate the efficacy of MRCSA in removing oscillatory peaks from the cross-spectrum. The robustness and precision of MRCSA is assessed by varying both the number and amplitude of oscillatory components, while the effect of noise on the performance of MRCSA is also explored. Finally, we describe the real-world datasets used for demonstration.

### The Self-Affine Property of Fractal Processes

Let’s consider a fractal process *f*(*t*) and its resampled version *f*_*h*_(*t*) = *f*(*t*/*h*), where *h* > 0 is the resampling factor. When *h* > 1 the process is ‘up-sampled’ and it is ‘down-sampled’ in case of 0 < *h* < 1. Illustratively, when *h=2* then *f*_*h*_(*t*) equals to *f*(*t*) sampled at twice its original sampling rate, while with *h* = 1/2 it is equal to taking only every second sample from *f*(*t*). Note that in this latter scenario *f*_*h*_(*t*) is referred to as the ‘coarse-grained’ version of *f*(*t*) by Yamamoto and Hughson (1993), see below. Then, the self-affine property of *f*(*t*) can be formalized as


(1) $$f_{h}\,\left(t\right)≜h^{H}\,f\,\left(t\right),$$

meaning that if *f*(*t*) is resampled by factor *h*, then the resampled time series *f*_*h*_(*t*) will have the same distribution as the original, only rescaled by factor *h^H^* with *H* termed the Hurst exponent (Mandelbrot and Van Ness, 1968; Yamamoto and Hughson, 1993; Eke et al., 2002). Importantly, when applying the Fourier transformation to *f*(*t*) and *f*_*h*_(*t*) this self-affine property can be readily established as the frequency scaling property


(2) $$F_{h}\,\left(\omega \right)≜h^{H}\,F\,\left(\omega \right),$$

where *F*(ω) and *F*_*h*_(ω) denotes the amplitudes of *f*(*t*) and *f*_*h*_(*t*) at angular frequency ω, respectively (the angular frequency ω relates to the sampling rate *r_s_* as ω = 2π*r*_*s*_). Relatedly, fractal processes are characterized by a continuous, broadband frequency distribution, where the spectral power (i.e., the squared amplitude) is inversely proportional to the frequency, and the relationship is established via a power-law function with scaling exponent β (Eke et al., 2002). When formalized as


(3) $${\left|F\,\left(\omega \right)\right|}^{2}\propto c\times \omega^{-\beta },$$

where *c* is a constant, it follows that the spectral power of a fractal signal is (theoretically) non-zero throughout the entire spectrum, as well as the spectrum follows a ‘straight line’ with slope −β when visualized on a log-log scale. It is important to highlight, that both *H* and capture the same scaling property of the process and thus they are equivalent and inherently related (Mandelbrot and Van Ness, 1968; Eke et al., 2000). In conclusion, the power (or amplitude) spectrum of a fractal process remains the same following resampling, only scaled by factor *h^H^*. This property is in sharp contrast with that of a periodic signal *x*(*t*) composed of a discrete set of sinusoidal components with characteristic frequencies ω_*i*_. In such a narrow-band signal the power spectrum is only non-zero at the specific frequencies corresponding to the constituting sinusoids, and zero (or close to zero) elsewhere. Importantly, in the power-spectrum of the resampled time series *x*_*h*_(*t*) these non-zero ‘peaks’ get relocated according to the resampling factor *h*, however, the spectral power remains zero elsewhere, including characteristic frequencies of the original signal (except for those cases where components ω_*i*_ and ω_*j*_ are related as ω_*i*_ = *h*×ω_*j*_). Exploiting this phenomenon offers means to decompose the power spectrum of a signal that is a mixture of fractal and oscillatory/periodic components, as detailed below.

### Extracting the Fractal Component of the Power Spectrum

#### Coarse Graining Spectral Analysis

The first method to separate the fractal component of a broadband spectrum was introduced by Yamamoto and Hughson (1991) for studying heart rate variability (HRV) time series. Interestingly, the aim of this approach was to prune the spectrum from the broadband fractal component and thus allowing for a better assessment of oscillatory peaks, which were in the center of interest regarding HRV studies (Yamamoto and Hughson, 1991). In this first approach the authors computed the cross-power spectrum of the original signal *X* with its coarse grained (i.e., resampled with *h* = 2^−1^) and rescaled (by dividing it by *h*^−*H*^) version *X_h_* to obtain the fractal component denoted as *S*_*XX_h_*_, which was then subtracted from the auto-power spectrum of the original signal, *S*_*XX*_ (Yamamoto and Hughson, 1991). This approach built on the aforementioned notions regarding Eq. (2), namely that (i) the amplitude spectra of a fractal process and its resampled and rescaled version should be the equivalent and thus their cross spectrum will be non-zero for all frequencies, while on the other hand (ii) the cross-spectrum of a harmonic signal with its resampled version will tend to zero for all frequencies due to the relocation of non-zero amplitudes in the spectra.

The method, though highly intuitive, suffers from multiple limitations, one of them being the fact that one had to first estimate *H* separately, which in this case was achieved by using rescaled range analysis (Mandelbrot and Wallis, 1969). This was later resolved by resampling the original process by factors of both *h* and its reciprocal 1/*h* (Yamamoto and Hughson, 1993). This way, one of the resampled versions is rescaled by *h^H^*, while the other by 1/*h^H^* = *h*^−*H*^ and thus if one takes the geometric mean of the two cross-spectra *S*_*XX_h_*_ and *S*_*XX_1/h_*_ as


(4) $${\bar{S}}_{XX_{h}}=\sqrt{\left\|S_{XX_{h}}\right\|\cdot \left\|S_{XX_{1/h}}\right\|},$$

where ${\bar{S}}_{X\,X_{h}}$ denotes the corrected fractal power spectrum, the computation no longer requires a separate estimation of *H*. Also note that this procedure is independent of the rescaling factor *h* > 0. A more severe limitation of the CGSA procedure, however, is the fact that the cross-spectrum of the original and the resampled version of a signal composed of fractal and oscillatory constituents will have non-negligible interactions between the two components that prevents the complete elimination of oscillatory peaks (Wen and Liu, 2016). Finally, CGSA can also break down in presence of multiple oscillatory components, when the characteristic frequencies of the oscillatory components are related as ω_*i*_ = *h*×ω_*j*_ or ω_*i*_ = 1/*h*×ω_*j*_.

#### Irregular-Resampling Auto-Spectral Analysis

IRASA was introduced recently by Wen and Liu (2016) to overcome these limitations. In contrast to CGSA, this approach explicitly assumes a simple additive model in which the investigated process *y*(*t*) is composed of a fractal *f*(*t*) and an oscillatory *x*(*t*) component without additive noise:


(5) y⁢(t)=f⁢(t)+x⁢(t).

According to the linearity property, applying the Fourier transform to *y*(*t*) yields


(6) $$Y\,\left(\omega \right)=F\,\left(\omega \right)\,e^{-j\,\alpha \,\left(\omega \right)}+X\,\left(\omega \right)\,e^{-j\,\beta \,\left(\omega \right)},$$

where *F*(ω) and α(ω) indicate the amplitude and phase of the fractal component at frequency ω, whlie *X*(ω) and β(ω) are the same terms regarding the oscillatory component. Then, let *y*_*h*_(*t*) and *y*_1/*h*_(*t*) denote the resampled versions of *y*(*t*) by factors *h* and 1/*h*, respectively (with *h* > 0). Using an analogous notation as introduced in Eq. (6), the auto-spectral power at frequency ω can be then defined as


(7) $$S_{y_{h}\,y_{h}}\,\left(\omega \right)=\left[F_{h}\,\left(\omega \right)\,e^{-j\,\alpha_{h}\,\left(\omega \right)}+X_{h}\,\left(\omega \right)\,e^{-j\,\beta_{h}\,\left(\omega \right)}\right]\,\left[F_{h}\,\left(\omega \right)\,e^{j\,\alpha_{h}\,\left(\omega \right)}+X_{h}\,\left(\omega \right)\,e^{j\,\beta_{h}\,\left(\omega \right)}\right]$$

for *y*_*h*_(*t*) and equivalently as


(8) $$S_{y_{1/h}\,y_{1/h}}\,\left(\omega \right)=\left[F_{1/h}\,\left(\omega \right)\,e^{-j\,\alpha_{1/h}\,\left(\omega \right)}+X_{1/h}\,\left(\omega \right)\,e^{-j\,\beta_{1/h}\,\left(\omega \right)}\right]\,\left[F_{1/h}\,\left(\omega \right)\,e^{j\,\alpha_{1/h}\,\left(\omega \right)}+X_{1/h}\,\left(\omega \right)\,e^{j\,\beta_{1/h}\,\left(\omega \right)}\right]$$

for *y*_1/*h*_(*t*). Then, by utilizing the notion Eq. (2) one can arrive on the forms


(9) $$S_{yhyh}\left(\text{ω}\right)=h^{2H}F^{2}\left(\text{ω}\right){\left\|1+\frac{X_{h}\left(\text{ω}\right)}{F_{h}\left(\text{ω}\right)}e^{j\alpha_{h}\left(\text{ω}\right)-j\beta_{h}\left(\text{ω}\right)}\right\|}^{2}$$

and


(10) $$S_{y1/hy1/h}\left(\text{ω}\right)=h^{-2H}F^{2}\left(\text{ω}\right){\left\|1+\frac{X_{1/h}\left(\text{ω}\right)}{F_{1/h}\left(\text{ω}\right)}e^{j\alpha_{1/h}\left(\text{ω}\right)-j\beta_{1/h}\left(\text{ω}\right)}\right\|}^{2}.$$

Then, one can simply take the geometric mean of the two auto-spectra equivalently to Eq. (4) to obtain an initial estimate of the fractal power spectrum, denoted as ${\bar{S}}_{h}\,\left(\omega \right)$, independent of *h* and *H* as


(11) $${\bar{S}}_{h}\left(\text{ω}\right)=\sqrt{S_{yhyh}\left(\text{ω}\right)S_{y1/hy1/h}\left(\text{ω}\right)}=F^{2}\left(\text{ω}\right)\left\|1+\frac{X_{h}\left(\text{ω}\right)}{F_{h}\left(\text{ω}\right)}e^{j\alpha_{h}\left(\text{ω}\right)-j\beta_{h}\left(\text{ω}\right)}\right\|\left\|1+\frac{X_{1/h}\left(\text{ω}\right)}{F_{1/h}\left(\text{ω}\right)}e^{j\alpha_{1/h}\left(\text{ω}\right)-j\beta_{1/h}\left(\text{ω}\right)}\right\|.$$

Let’s assume the oscillatory component *x*(*t*) consists of a single sinusoid at harmonic frequency ω_0_. From Eq. (11) it then follows, that ${\bar{S}}_{h}\,\left(\omega \right)\,F^{2}\,\left(\omega \right)$ in only two cases:


(12) $$\left(a\right)\;{\bar{S}}_{h}\left(\text{ω}\right)=F^{2}\left(\text{ω}\right)\left\|1+\frac{X_{h}\left(\text{ω}\right)}{F_{h}\left(\text{ω}\right)}e^{j\alpha_{h}\left(\text{ω}\right)-j\beta_{h}\left(\text{ω}\right)}\right\|\text{ifω}=h{\text{ω}}_{0}\;\mathrm{and}$$

$$\left(b\right)\;{\bar{S}}_{h}\left(\text{ω}\right)=F^{2}\left(\text{ω}\right)\left\|1+\frac{X_{1/h}\left(\text{ω}\right)}{F_{1/h}\left(\text{ω}\right)}e^{j\alpha_{1/h}\left(\text{ω}\right)-j\beta_{1/h}\left(\text{ω}\right)}\right\|\text{ifω}=h{\text{ω}}_{0}/h$$

Note, however, that these instances where ${\bar{S}}_{h}\,\left(\omega \right)$ does not yield an unbiased estimate of the fractal spectrum always depend on *h*; with using multiple distinct resampling factors, the non-zero oscillatory power at ω_0_ gets recolated to a different frequency with each value. Consequently, carrying out the estimation procedure using not only one but instead a set of non-integer resampling factors (and their respective reciprocals) yields a population of fractal spectral estimates for all frequencies that mostly center around the true *F*^2^(ω), except if one of the scenarios in Eq. (12) holds, as in that case there will be typically one outlier at estimate for the corresponding *h*-value. Therefore, taking the median among these estimates at each individual frequency will yield an unbiased estimate of *F*^2^(ω) for all ω as long as the number of outliers does not surpass the number of estimates (Wen and Liu, 2016). Notably, using a sufficiently large number (e.g., >15) of different *h* values also renders IRASA robust against the presence of multiple oscillatory components by reducing the probability of too many of them being interrelated as ω_*i*_ = *h*×ω_*j*_ or ω_*i*_ = 1/*h*×ω_*j*_.

Additionally, the power spectrum of the composite signal of Eq. (5) is defined as


(13) $$Y^{2}\,\left(\omega \right)=Y\,\left(\omega \right)\,\bar{Y\,\left(\omega \right)}=F^{2}\,\left(\omega \right)+X^{2}\,\left(\omega \right)+2\,F\,\left(\omega \right)\,X\,\left(\omega \right)\,\cos\left(\alpha \,\left(\omega \right)-\beta \,\left(\omega \right)\right).$$

where $\bar{Y\,\left(\omega \right)}$ denotes the complex conjugate of *Y*(ω). Eq. (13) shows that the mixed power spectrum is composed not only of the spectral densities of the fractal and oscillatory components but also a confounding term whose magnitude depends on the phase difference between fractal and oscillatory components. However, by assuming no coupling between the two components the expectation of the second term is zero and thus the confounding term can be eliminated by computing the power spectrum from multiple data segments and then taking the average over the obtained spectra. Note that this also assumes that segments were obtained from a period during which the process is stationary. Finally, one can obtain a theoretically unbiased estimate of the oscillatory power spectrum by subtracting the fractal from the mixed spectrum (Wen and Liu, 2016).

### Multiple-Resampling Cross-Spectral Analysis

Podobnik and Stanley (2008) introduced the first method to assess long-range fractal coupling between two (non-stationary) processes, termed detrended cross-correlation analysis (DCCA). DCCA was quickly generalized to the multifractal domain (Zhou, 2008), while also complemented by other time-domain methods such as the detrended moving-average cross-correlation analysis (Arianos and Carbone, 2009) or the height cross-correlation analysis (Kristoufek, 2011). Similarly to the univariate case, bivariate fractal scaling of processes *x* and *y* is captured in the bivariate Hurst exponent *H*_*xy*_ (Kristoufek, 2011). Moreover, this property can be equivalently captured in the frequency domain as well (Kristoufek, 2014). Precisely, in case of long-term fractal coupling between two processes *x* and *y*, cross-spectral power *SS*_*xy*_(ω) is proportional to the frequency ω via a power-law function with cross-spectral exponent β_*xy*_ as


(14) $$\left|S\,S_{x\,y}\right|\,\left(\omega \right)\propto c\times \omega^{-\beta_{x\,y}}.$$

This case is analogous with that defined for the univariate case in Eq. (3), as well as there is an exact correspondence between *H*_*xy*_ and β_*xy*_ (Kristoufek, 2014, 2017). Similarly, the frequency scaling property also holds; let’s define bivariate fractal processes *k*(*t*) and *l*(*t*) with bivariate Hurst exponent *H*_*kl*_. Then, following the resampling both processes by factor *h*, their cross-spectrum *SS*_*k*_*h*_*l*_*h*__(ω) will be equivalent to the cross-spectrum *SS*_*kl*_(ω) of the original processes rescaled by *h^H^*_*kl*_:


(15) $$\left|S\,S_{k_{h}\,l_{h}}\,\left(\omega \right)\right|≜h^{H_{k\,l}}\,\left|S\,S_{k\,l}\,\left(\omega \right)\right|.$$

This can be shown when applying the formula from Eq. (13) to obtain the cross-spectrum:


(16) $$S\,S_{k_{h}\,l_{h}}\,\left(\omega \right)=F_{k_{h}}\,\left(\omega \right)\,\bar{F_{l_{h}}\,\left(\omega \right)}=h^{H_{k}}\,F_{k}\,\left(\omega \right)\,\bar{h^{H_{l}}\,F_{l}\,\left(\omega \right)}=h^{H_{k}+H_{l}}\,F_{k}\,\left(\omega \right)\,\bar{F_{l}\,\left(\omega \right)}=h^{2\,H_{k\,l}}\,S\,S_{k\,l}\,\left(\omega \right).$$

From Eqs. (15) and (16) it also follows automatically that $H_{x\,y}=\frac{H_{x}+H_{y}}{2}$, as observed in many previous studies from both theoretical and simulation standpoints (Podobnik and Stanley, 2008; Podobnik et al., 2011; Kristoufek, 2013a,b). Note, however, that processes with $H_{x\,y}<\frac{H_{x}+H_{y}}{2}$ have also been proposed (Sela and Hurvich, 2012; Kristoufek, 2013a), as their existence are theoretically plausible, unlike those with $H_{x\,y}>\frac{H_{x}+H_{y}}{2}$ (Kristoufek, 2015). Therefore, along the same lines of thinking as with Eqs. (7)–(12), one can exploit this property and construct a method to separate the fractal component of the cross-spectrum.

**FIGURE 1:**
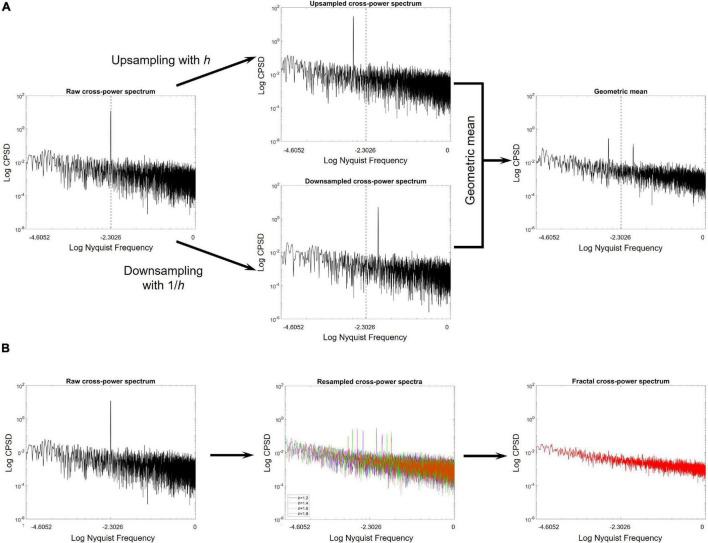
Key steps of the MRCSA procedure. **(A)** The cross-power spectrum is shown on the left, obtained from a pair of long-range cross-correlated time series with a strongly correlated oscillatory component at 10% of the Nyquist frequency. The middle panels show the cross-power spectra obtained after upsampling (upper) and downsampling (lower) the pair of signals by factors *h* and 1/*h*, respectively. It is clearly observable, that resampling relocates the oscillatory peak from its ‘original position’ in both cases. The right panel shows the geometric mean of the up- and downsampled cross-spectra. **(B)** On the left, the raw cross-power spectrum is illustrated. Geometric means of the up- and downsampled cross-spectra are obtained after resampling with various values of *h* (middle). Finally, by taking the median one can obtain the fractal cross-power spectrum, with no oscillatory peak.

The key steps of MRCSA are illustrated on Figure 1. More formally, let’s assume two processes, *x*(*t*) = *f*_*x*_(*t*) + *h*_*x*_(*t*) and *y*(*t*) = *f*_*y*_(*t*) + *h*_*y*_(*t*), both composed as a mixture of fractal and oscillatory (harmonic) components and with Hurst exponents *H_x_* and *H_y_*. Generally, the cross-power spectrum of *x*(*t*) and *y*(*t*), denoted as |*SS*_*xy*_(ω)| can be obtained as


(17) $$\left|S\,S_{x\,y}\,\left(\omega \right)\right|=\left|F_{x}\,\left(\omega \right)\,\bar{F_{y}\,\left(\omega \right)}\right|,$$

where *F*_*x*_(ω) and *F*_*y*_(ω) mark the Fourier transforms of *x*(*t*) and *y*(*t*), respectively. After resampling with factor *h* and 1/*h* we obtain the resampled time series *x*_*h*_(*t*), *y*_*h*_(*t*), *x*_1/*h*_(*t*) and *y*_1/*h*_(*t*). Let *FX*_*h*_(ω)*e*^−*j*α_*h*_(ω)^ and *FY*_*h*_(ω)*e*^−*j*γ_*h*_(ω)^ denote the Fourier transforms of the fractal, while *HX*_*h*_(ω)*e*^−*j*β_*h*_(ω)^ and *HY*_*h*_(ω)*e*^−*j*δ_*h*_(ω)^ the Fourier transforms of the oscillatory (harmonic) components of series *x*_*h*_(*t*) and *y*_*h*_(*t*), respectively [terms for *x*_1/*h*_(*t*) and *y*_1/*h*_(*t*) are defined analogously]. Then, the cross-power spectra of *x*_*h*_(*t*) and *y*_*h*_(*t*) can be obtained as


(18) $$S\,S_{x_{h}\,y_{h}}\,\left(\omega \right)=\left[F\,X_{h}\,\left(\omega \right)\,e^{-j\,\alpha_{h}\,\left(\omega \right)}+H\,X_{h}\,\left(\omega \right)\,e^{-j\,\beta_{h}\,\left(\omega \right)}\right]\left[F\,Y_{h}\,\left(\omega \right)\,e^{j\,\gamma_{h}\,\left(\omega \right)}+H\,Y_{h}\,\left(\omega \right)\,e^{j\,\delta_{h}\,\left(\omega \right)}\right]==h^{H_{x}+H_{y}}\,F\,X\,\left(\omega \right)\,F\,Y\,\left(\omega \right)\,e^{-j\,\left(\alpha_{h}\,\left(\omega \right)-\gamma_{h}\,\left(\omega \right)\right)}\left(1+\frac{H\,X_{h}\,\left(\omega \right)}{F\,X_{h}\,\left(\omega \right)}\,e^{-j\,\left(\alpha_{h}\,\left(\omega \right)-\beta_{h}\,\left(\omega \right)\right)}\right)\,\left(1+\frac{H\,Y_{h}\,\left(\omega \right)}{F\,Y_{h}\,\left(\omega \right)}\,e^{j\,\left(\gamma_{h}\,\left(\omega \right)-\delta_{h}\,\left(\omega \right)\right)}\right)$$

and similarly, for *x*_1/*h*_(*t*) and *y*_1/*h*_(*t*) as


(19) $$S\,S_{x_{1/h}\,y_{1/h}}\,\left(\omega \right)=\left[F\,X_{1/h}\,\left(\omega \right)\,e^{-j\,\alpha_{1/h}\,\left(\omega \right)}+H\,X_{1/h}\,\left(\omega \right)\,e^{-j\,\beta_{1/h}\,\left(\omega \right)}\right]\left[F\,Y_{1/h}\,\left(\omega \right)\,e^{j\,\gamma_{1/h}\,\left(\omega \right)}+H\,Y_{1/h}\,\left(\omega \right)\,e^{j\,\delta_{1/h}\,\left(\omega \right)}\right]==h^{-\left(H_{x}+H_{y}\right)}\,F\,X\,\left(\omega \right)\,F\,Y\,\left(\omega \right)\,e^{-j\,\left(\alpha_{1/h}\,\left(\omega \right)-\gamma_{1/h}\,\left(\omega \right)\right)}\left(1+\frac{H\,X_{1/h}\,\left(\omega \right)}{F\,X_{1/h}\,\left(\omega \right)}\,e^{-j\,\left(\alpha_{1/h}\,\left(\omega \right)-\beta_{1/h}\,\left(\omega \right)\right)}\right)\left(1+\frac{H\,Y_{1/h}\,\left(\omega \right)}{F\,Y_{1/h}\,\left(\omega \right)}\,e^{j\,\left(\gamma_{1/h}\,\left(\omega \right)-\delta_{1/h}\,\left(\omega \right)\right)}\right).$$

One can then obtain an estimate of the fractal cross-power spectrum, denoted ${\bar{S\,S}}_{h}\,\left(\omega \right)$, by computing the geometric mean of ||*S*_*x*_*h*_*y*_*h*__(ω)|| and *S*_*x_1/h_y_1/h_*_:


(20) $$\bar{S}{\bar{S}}_{h}\left(\text{ω}\right)=\sqrt{\left\|S_{xhyh}\right\|\left\|\left(\text{ω}\right)S_{x1/hy1/h}\left(\text{ω}\right)\right\|}=\left|F_{X}\left(\text{ω}\right)F_{Y}\left(\text{ω}\right)\right|\sqrt{\left\|1+A_{h}\left(\text{ω}\right)\right\|\left\|1+B_{h}\left(\text{ω}\right)\right\|\left\|1+C_{1/h}\left(\text{ω}\right)\right\|\left\|1+D_{1/h}\left(\text{ω}\right)\right\|},$$

where

• $A_{h}\,\left(\omega \right)=\frac{H\,X_{h}\,\left(\omega \right)}{F\,X_{h}\,\left(\omega \right)}\,e^{-j\,\left(\alpha_{h}\,\left(\omega \right)-\beta_{h}\,\left(\omega \right)\right)}$,
• $B_{h}\,\left(\omega \right)=\frac{H\,Y_{h}\,\left(\omega \right)}{F\,Y_{h}\,\left(\omega \right)}\,e^{j\,\left(\gamma_{h}\,\left(\omega \right)-\delta_{h}\,\left(\omega \right)\right)}$,
• $C_{1/h}\,\left(\omega \right)=\frac{H\,X_{1/h}\,\left(\omega \right)}{F\,X_{1/h}\,\left(\omega \right)}\,e^{-j\,\left(\alpha_{1/h}\,\left(\omega \right)-\beta_{1/h}\,\left(\omega \right)\right)}$ and
• $D_{1/h}\,\left(\omega \right)=\frac{H\,Y_{1/h}\,\left(\omega \right)}{F\,Y_{1/h}\,\left(\omega \right)}\,e^{j\,\left(\gamma_{1/h}\,\left(\omega \right)-\delta_{1/h}\,\left(\omega \right)\right)}$.

Note that the above terms *A*(ω) to *D*(ω) capture the relationships between the fractal and oscillatory components of the processes in terms of the ratio of magnitudes and the difference in phases. Considering Eq. (20), one can draw similar conclusions as in case of IRASA (Wen and Liu, 2016), namely that

(i) If *x*(*t*) and *y*(*t*) only consist of fractal components, then ${\bar{S\,S}}_{h}\,\left(\omega \right)$ is always equal to the fractal cross-power spectrum and thus unbiased, as all the confounding terms are equal to zero at all ω.
(ii) If *x*(*t*) contains harmonic component with characteristic frequency ω_*HX*_, then the term *A*_*h*_(ω) will be non-zero if ω_1_ = *h*ω_*HX*_ and the term *C*_1/*h*_(ω) will be non-zero if ω_2_ = *h*/ω_*HX*_ and thus ${\bar{S\,S}}_{h}\,\left(\omega \right)$ will be biased at ω_1_ and ω_2_.
(iii) If *y*(*t*) contains harmonic component with characteristic frequency ω_*HY*_, then the term *B*_*h*_(ω) will be non-zero if ω_3_ = *h*ω_*HY*_ and the term *D*_1/*h*_(ω) will be non-zero if ω_4_ = *h*/ω_*HX*_ and thus ${\bar{S\,S}}_{h}\,\left(\omega \right)$ will be biased at ω_3_ and ω_4_.

The above described procedure indicates that ${\bar{S\,S}}_{h}\,\left(\omega \right)$ is an unbiased estimate of the fractal cross-power spectrum except for cases (ii) and (iii), which are all dependent on the rescaling factor *h*. Consequently, if one computes ${\bar{S\,S}}_{h}\,\left(\omega \right)$ using different values of *h*, the frequencies where the estimation errors occur will also be different. Therefore, if one obtains a population of ${\bar{S\,S}}_{h}\,\left(\omega \right)$ estimates, each calculated with different *h* value, then by taking the median over these for every frequency ω will yield an unbiased estimate of the fractal cross-power spectrum as long as the number of outliers (i.e., the occurring estimation errors) at the given frequency is less than 50% (Bassett, 1991; Wen and Liu, 2016). Therefore, we finally arrive at the formula for the unbiased fractal cross-power spectrum *SF*_*XY*_(ω):


(21) $$S\,F_{X\,Y}\,\left(\omega \right)=m\,e\,d\,i\,a\,n_{h}\,\left\{{\bar{S\,S}}_{h}\,\left(\omega \right)\right\},$$

for each ω.

Finally, one might desire to obtain an unbiased separate estimate of the oscillatory cross-spectrum, *SH*_*XY*_(ω) as well. However, if one computes |*SS*_*XY*_(ω)|^2^ analogously to Eq. (13), it can be shown that the cross-spectral power is related not only to *SF*_*XY*_(ω), *SH*_*XY*_(ω) and confounding terms depending on the relative phase difference between the fractal and oscillatory components of each process (separately), but also on interaction terms between the fractal component of *x*(*t*) and the oscillatory component of *y*(*t*), and vice versa (for details, see Supplementary Material). Assuming no coupling between *f*_*x*_(*t*) and *h*_*x*_(*t*) [and similarly for *f*_*y*_(*t*) and *h*_*y*_(*t*)] – and thus an even random distribution of relative phase differences – the former confounding terms can be eliminated by taking multiple data segments and averaging the obtained cross-power spectra. However, those terms capturing the interaction between *f*_*x*_(*t*) and *h*_*y*_(*t*) [and similarly, between *f*_*y*_(*t*) and *h*_*x*_(*t*)] do not depend on the relative phase difference and thus cannot be eliminated by averaging. Therefore, MRCSA by itself cannot provide an unbiased estimate on the oscillatory cross-power spectrum, only on the fractal cross-power spectrum. Nevertheless, an upper bound to the contribution of oscillatory components in the cross-spectrum can be obtained by computing the percentage of fractal cross-spectral power to the full (mixed) cross-spectral power as


(22) $$\%Fractal=\frac{\sum_{\omega }S\,F_{X\,Y}\,\left(\omega \right)}{\sum_{\omega }S\,S_{X\,Y}\,\left(\omega \right)}\times 100,$$

where the sum runs over all frequencies ω.

There are two important notions to mention. First, from Eqs. (17)–(21) it can be seen that by making *y*(*t*) equal to *x*(*t*), the MRCSA method reduces to simple IRASA. Secondly, if one computes not only the fractal cross-spectral power between *x*(*t*) and *y*(*t*) but also their fractal and oscillatory spectra separately with IRASA, the remaining confounding interaction terms from |*SS*_*XY*_(ùù)|^2^ can also be eliminated and thus a theoretically unbiased estimate of oscillatory cross-spectral power can also be obtained (see Supplementary Material). Nevertheless, the primary focus of this paper is to provide an unbiased estimate of the fractal cross-spectral power, and thus this issue is not discussed here further.

#### The Multiple-Resampling Cross-Spectral Analysis Algorithm

In order to manage consistency among the uni- and the bivariate cases, the MRCSA algorithm follows the approach presented for IRASA by Wen and Liu (2016).

(i) From the given pair of signals to be analyzed fifteen partially overlapping segments are selected (with equal time stamps), each with 90% length of the entire original datasets. The data segments are evenly distributed, i.e., the difference between their starting time indices is constant.
(ii) For the first data segment as defined above the native (mixed) cross-power spectrum *SS*_*xy*_(ω) is estimated according to Eq. (17), where the Fourier transforms are obtained using fast Fourier transform and Hanning windowing. Frequency resolution is set to be twice as the smallest power of two that exceeds the number of data points in the time segments (achieved by zero padding of the time series, when necessary). This is to ensure that in case of *h* < 2 the number of frequencies are greater than the number of data points both in the original signal and its resampled versions (see below).
(iii) The data segments are then resampled by *h* and 1/*h* using cubic spline interpolation. In case of downsampling the data segments are low-pass filtered with a fast Fourier transform-based filter to avoid aliasing (with the cutoff frequency defined as the sampling rate divided by twice the smallest integer larger than the largest *h*-value). Similarly to IRASA, we set *h* by default to range from 1.1 to 1.9 with 0.05 increments, resulting in 17 different resampling factor pairs.
(iv) Cross-power spectra for the up- and downsampled signal segment pairs are obtained using the similar procedure as described in step (ii). Importantly, the frequency resolution is kept the same as for the native cross-power spectrum for both the up- and downsampled signal segments (with appropriate zero-padding).
(v) The geometric mean of the cross-power spectra is obtained for all {*h*|1/*h*} pairs. Then for every frequency the median of the cross-power spectra over all *h* is taken to yield the unbiased estimate of the fractal cross-power spectrum, *SF*_*XY*_(ω).
  (vi) Steps (ii)–(v) are repeated for all data segments obtained from step (i), and then the average of both *SS*_*xy*_(ω) and *SF*_*xy*_(ω) are computed by taking the arithmetic mean over the cross-spectra obtained from the 15 data segments.

After the MRCSA pipeline is completed, one can proceed to obtain the cross-spectral slope, β_*xy*_ and the percentage of fractal cross-spectral power. The spectral slope can be acquired by fitting a linear function on the log-log transformed fractal cross-power spectrum. However, this procedure in itself would result in an increasing over-representation of higher frequency components due to the log transformation (Wen and Liu, 2016). Therefore, frequency components are first resampled following the log transform to yield an even frequency resolution. Then, a linear function is fitted on the resampled fractal cross-power spectrum by ordinary least squares estimation, and the β_*xy*_ is obtained as the first coefficient (slope) of the function. Note that in general fractal spectral slope is negative (i.e., the cross-spectral power follows a 1/ω^β_*xy*_^ distribution), however, according to convention we report β_*xy*_ values with reversed signs similarly to univariate spectral exponents (Eke et al., 2002), so that a steeper cross-spectrum is characterized with a larger positive β_*xy*_ value. The percentage of fractal cross-spectral power is obtained simply by applying Eq. (22) in the selected frequency range of interest. Note that similarly to IRASA, MRCSA can also be utilized in a sliding-window manner (Wen and Liu, 2016) to provide a time-frequency representation of fractal cross-spectral power between a longer period of two coupled processes.

A Matlab implementation of the MRCSA algorithm will be made available soon at the repository at http://github.com/samuelracz/MRCSA. All simulations and data analyses described below were carried out using Matlab (The Mathworks, Natick, MA, United States).

### *In silico* Experiments and Evaluation

Multiple-resampling cross-spectral analysis was first tested on simulated time series with known cross-spectral slope. For the simulation purpose we adopted the framework of mixed-correlated ARFIMA(0,*d*,0) (autoregressive fractionally integrated moving average) processes introduced by Kristoufek (2013a) [in the following we will omit the (0,*d*,0) specification for the sake of simplicity]. With appropriate parametrization a mixed-correlated ARFIMA process – which consists of a pair of long-range correlated time series – has known bivariate Hurst exponent, that is not necessarily the average of the univariate Hurst exponents of the constituting time series. Generally, an ARFIMA process *p_t_* can be defined as


(23) $$p_{t}=\sum_{n=0}^{+\infty }a_{n}\,\left(d\right)\,\varepsilon_{t-n}$$

where *d* ∈ [0, 0.5) is the scaling parameter *n* is the time scale, ε_*t*_ denotes independent and identically distributed Gaussian random variables with zero mean and unit variance, and weights *a*_*n*_(*d*) are defined as $a_{n}\,\left(d\right)=\frac{d\,\Gamma \,\left(n+d\right)}{\Gamma \,\left(d\right)\,\Gamma \,\left(n+1\right)}$ where Γ denotes the Gamma function (Podobnik et al., 2008). An ARFIMA process has fractal scaling with its Hurst exponent *H* = 0.5 + *d* (note that in case of *d=0* the process reduces to white noise). A mixed-correlated ARFIMA process (Kristoufek, 2013a) is in fact a bivariate time series, where each of its constituents is a combination of two ARFIMA processes so that


$$u_{t}=w_{1}\,\sum_{n=0}^{+\infty }a_{n}\,\left(d_{1}\right)\,\varepsilon_{1,t-n}+w_{2}\,\sum_{n=1}^{+\infty }a_{n}\,\left(d_{2}\right)\,\varepsilon_{2,t-n}$$

(24) $$v_{t}=w_{3}\,\sum_{n=0}^{+\infty }a_{n}\,\left(d_{3}\right)\,\varepsilon_{3,t-n}+w_{4}\,\sum_{n=0}^{+\infty }a_{n}\,\left(d_{4}\right)\,\varepsilon_{4,t-n}$$

where


$$\left\langle \varepsilon_{i,t}\right\rangle =0\,\text{for}\,i=1,2,3,4$$

$$\left\langle \varepsilon_{i,t}^{2}\right\rangle =\sigma_{\varepsilon_{i}}^{2}\,\text{for}\,i=1,2,3,4$$

$$\left\langle \varepsilon_{i,t}\,\varepsilon_{j,t-n}\right\rangle =0\,\text{for}\,n\neq 0\,\text{and}\,i,j=1,2,3,4$$

$$\left\langle \varepsilon_{i,t}\,\varepsilon_{j,t}\right\rangle =\rho_{i\,j}\,\text{for}\,i,j=1,2,3,4\,r\,m\,a\,n\,d\,i\neq j.$$

In words, the each of the two processes is itself a linear combinations of two separate ARFIMA processes with weights *w*_*i*_,*i* = 1,2,3,4, Hurst exponents *H*_*i*_,*i* = 1,2,3,4 (defined by parameters *d*_*i*_,*i* = 1,2,3,4 as *H*_*i*_ = *d*_*i*_ + 0.5), and innovations (ε_*i*,*t*_) that are may or may not be correlated. It can be shown that with appropriate parametrization *u_t_* and *v_t_* are long-long range cross-correlated (i.e., they have a fractal cross-power spectrum) with bivariate Hurst exponent $H_{u\,v}=\frac{H_{2}+H_{3}}{2}$ (Kristoufek, 2013a). Given that |*SS*_*xy*_(ω) |αω^1−2*H*_*xy*_^ (Kristoufek, 2014), one can conclude that the cross-spectral power of *u_t_* and *v_t_* scales as β_*uv*_ = *H*_2_ + *H*_3_1−(0.5 + *d*_2_) + (0.5 + *d*_3_)−1 = *d*_2_*d*_3_. The true presence of long-range cross-correlations can be ensured by setting ρ_2,3_ > 0 with leaving ρ_*ij*_ = 0 for the rest of the cases. Finally, correlated oscillatory components can be introduced to the model by simply adding the same sinusoidal signal to both *u_t_* and *v_t_*.

For testing the precision and robustness of MRCSA against the presence of correlated oscillatory components varying in their number and amplitude, we simulated *n=100* time series pairs for each case of parameter combinations. Time series were simulated at 500 Hz sampling rate and with length 10,000 data points. Sinusoids were introduced in the following manner. First, the generated mixed-correlated ARFIMA time series pairs were standardized to have zero mean and unit variance. Then, sinusoidal signals of equal length and desired frequency ω_*i*_ (with *i* ranging from 1 to the number of oscillatory components) were generated and standardized to unit variance. Their variance was then set to a desired proportion (ranging from 0.16 to 5.12) and scaled down by a factor of $\omega_{i}^{\beta_{u\,v}}$ so to adjust to the cross-spectral fractal power at the same frequency. The number of sinusoidal components were varied from 1 to 7, while their amplitudes (variances) were varied from 16 to 512% of that of the ARFIMA signal in dyadic increments. Following recommendations of Kristoufek (2013a), the mixed-correlated ARFIMA time series were generated with parameter settings *w*_1_ = *w*_4_ = 0.1, *w*_2_ = *w*_3_ = 1, *d*_1_ = 0.4, *d*_2_ = 0.3, *d*_3_ = 0.2, *d*_4_ = 0.3, $\sigma_{\varepsilon_{i}}^{2}=1$ for *i* = 1,2,3,4 and ρ_2,3_ = 0.9 (with ρ_*i*,*j*_ = 0 in all other cases) to highlight the cross persistence between the two time series. From the parameter settings it follows that the generated time series had a theoretical β_*uv*_ = 0.5. The phases of the added sinusoidals were drawn from a uniform random distribution.

We evaluated the efficacy of MRCSA by computing the difference of cross-spectral exponents and percentage of fractal cross-power obtained from the mixed and the separated fractal cross-spectra (from here on denoted as _*mixed*_ and _*fractal*_, respectively). Kristoufek (2014) showed that spectrum-based estimators of the bivariate Hurst exponent (including the cross-periodogram estimator, which is highly similar to MRCSA) are biased to varying extent when tested in a mixed-correlated ARFIMA framework. Therefore, instead of comparing the obtained cross-spectral slopes to their corresponding expected theoretical value, we computed the difference of _*mixed*_ and _*fractal*_ to the spectral exponent derived via MRCSA from the raw mixed-correlated ARFIMA time series pairs, i.e., before the addition of the sinusoidal components (denoted as _*raw*_).

We also assessed the effect of white noise on the performance of MRCSA. We simulated mixed-correlated ARFIMA processes with the same parameter settings as defined above (i.e., _*uv*_ = 0.5) and introduced a single oscillatory component at 10 Hz. Then, we added random, uncorrelated noise components to both time series with signal to noise ratio (SNR) of 0, 1, 10, and 100%. Following Wen and Liu (2016), SNR was defined as the ratio of the total variance of the signal and the total variance of the additive noise, converted to percentage.

### Experimental Data

We also demonstrate the applicability of MRCSA on real-world neurophysiological data. For this purpose, we used EEG recordings of an openly available database (Shin et al., 2018) obtained in baseline (resting-state) and cognitive stimulation (word generation) conditions. For the sake of simplicity here we only provide a brief description of the data, for further details the reader is referred to the original publication of Shin et al. (2018).

#### Subjects, Experimental Paradigm and Data Acquisition

Data were obtained from 26 young, healthy volunteers (17 females, aged 26.1 ± 3.5 years, all right-handed). None of the participants had any history of neuropsychological pathology. The experiment was carried out in line with the Declaration of Helsinki, all volunteers provided written informed consent before the recording, and the original study was approved by the regional ethical committee at the Berlin Institute of Technology (approval number: SH_01_20150330). Further details on the participants are found in the Supplementary Information of Shin et al. (2018).

Participants were seated in a comfortable armchair in front of an LCD monitor. The total experimental paradigm consisted of three different cognitive tasks, however, for this demonstration we only used data from Dataset C, the word generation (WG) paradigm. In this paradigm, participants were presented an initial letter at every test trial, and their task was to think of as many different words as they can starting with the given letter. Contrary, in the baseline trials they were only presented a fixation cross, and they were instructed to relax. Both the presentation and baseline (BL) trials were of duration 10 s, interspersed with relaxation periods varying in length (∼20 s in total for each trial). Each participant completed three separate sessions, and each session consisted of 10–10 BL and WG trials in randomized order. Thus, every participant completed 30 BL and 30 WG trials in total (each lasting for 10 s). A more detailed description of the cognitive paradigm is illustrated on Figure 1 of Shin et al. (2018) and the related text.

The EEG data was recorded using a BrainAmp EEG amplifier (Brain Products GmbH, Gilching, Germany) from 28 cortical regions (Fp1, Fp2, AFF5h, AFF6h, AFz, F1, F2, FC1, FC2, FC5, FC6, Cz, C3, C4, T7, T8, CP1, CP2, CP5, CP6, Pz, P3, P4, P7, P8, POz, O1, and O2) according to the international 10–5 system (Oostenveld and Praamstra, 2001). Reference and ground electrodes were located at TP9 and TP10, respectively. Sampling rate was set to 1,000 Hz, however, the data was downsampled to 200 Hz before made publicly available.

#### Data Pre-processing and Analysis

Electroencephalography preprocessing was carried out using the EEGLAB toolbox (Delorme and Makeig, 2004) along with custom functions and scripts. First, the 10-s BL and WG periods were isolated for each subject. For preprocessing purposes (see below), 5 s preceding and 15 s following the 10-s periods (both relaxation periods) were also included in these initial data segments. Data was then band-pass filtered between 0.5 and 80 Hz with a 4th order zero-phase Butterworth filter. Additional line noise removal was performed at 50Hz with the *cleanline* algorithm as implemented in EEGLAB. Automated artifact identification was carried out using independent component analysis (ICA) combined with multiple artifact rejection algorithm (MARA) (Winkler et al., 2011, 2014). Data was then re-referenced to the common average reference and baseline corrected using the average of the 5 s data preceding the BL/WG trial periods. Finally, the 10-s BL and WG periods were isolated for further analysis (30 + 30 segments per subject).

The MRCSA was used to compute the fractal cross-power spectrum between all channel pairs for all data segments. MRCSA was also used to recover the fractal auto-power spectrum of all individual channels by feeding the same time series as both inputs. Auto- and cross-spectral exponents were obtained from the separated fractal spectra as described previously and organized into a three-dimensional matrices of size 28 channels × 28 channels × 30 trials for BL and WG, for each subject. Percentage of fractal power was estimated and sorted similarly. Finally, matrices obtained from different trials under the same conditions were averaged so to provide a robust estimate of the characteristic activity pattern of the condition for each subject.

We compared the obtained measures between BL and WG states in four different manners: (i) we compared auto-spectral exponents at every channel, (ii) the sum of cross-spectral exponents for each channel – which can be considered as the ‘node degree’ of the given location in graph theoretical terms (Rubinov and Sporns, 2010) –, (iii) the cross-spectral exponents individual connections separately, and (iv) the percentage of fractal cross-spectral power of all individual connections. Normal distribution of data was assessed with Lilliefors tests. Values from the two conditions were compared using paired *t*-tests in case of normality, while using paired Wilcoxon signed rank tests otherwise. Initial level of significance was defined at *p* < 0.05, which was then adjusted for multiple comarisons in each case (i)–(iv) with the Bonferroni method.

## Results

### *In silico* Experiments

#### Demonstration

**FIGURE 2:**
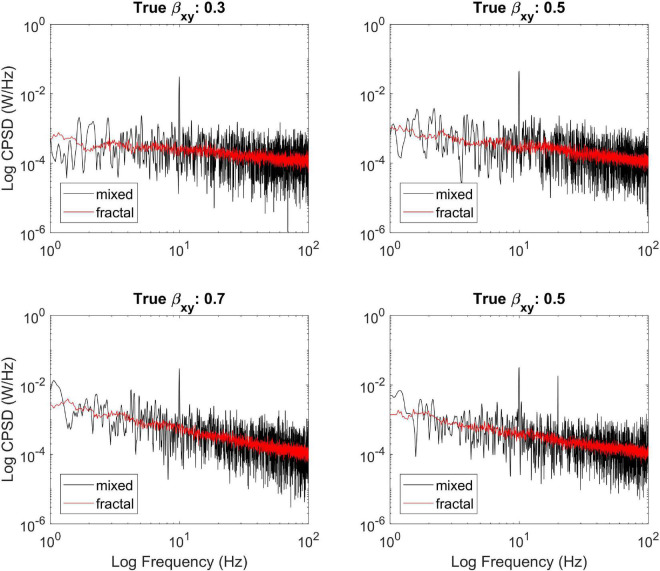
Illustrating the efficiency of MRCSA at different theoretical values of β_*xy*_. It can be seen that MRCSA efficiently eliminated the oscillatory peak in all cases **(Upper,Lower left)**, as well as it was unaffected by the presence of multiple oscillatory peaks **(Lower right)**.

First, we illustrate the ability of MRCSA to remove oscillatory components from the cross-power spectrum (Figure 2). We simulated three different mixed-correlated ARMIFA processes with theoretical β_*xy*_ values of 0.3, 0.5, and 0.7 and introduced a correlated sinusoidal component into each of them at 10 Hz, as well as another bivariate process with theoretical β_*xy*_ = 0.5 which was mixed with two harmonic signals at 10 and 20 Hz. It is clearly visible that MRCSA succeeds in eliminating the peak in cross-spectral density at 10 Hz, as well as its performance remains steady with two instead of one oscillatory peak.

#### Effect of Oscillation Number and Amplitude

**FIGURE 3:**
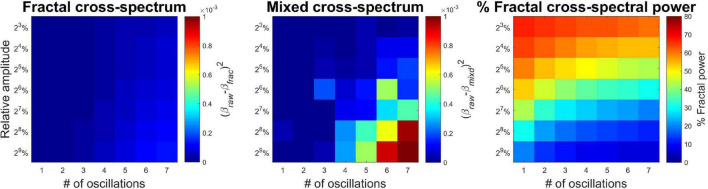
Precision of MRCSA in the presence of oscillatory components of varying number and amplitude. The **(Left)** shows squared differences between β_*xy*_ when obtained from the fractal cross-spectrum and the cross-spectrum of raw mixed-correlated ARFIMA processes (without oscillatory components). The **(Middle)** shows the same difference, only β_*xy*_ was estimated from the raw cross-spectrum (and not the separated fractal cross-spectrum). The **(Right)** shows the percentage of fractal cross-spectral power.

Performance of MRCSA under varying conditions is illustrated on Figure 3. The left panel clearly shows that MRCSA yielded estimates almost equal to those obtained from pure mixed-correlated ARFIMA processes without oscillatory components. There is a very weak increase in squared difference with increasing both the number and amplitude of oscillatory components. In contrast, the squared errors became larger by a magnitude under similar conditions when computing the cross-spectral slope from the mixed cross-coherence spectrum (middle panel). These results imply that under realistic conditions relevant to neurophysiological signals (small number of oscillations, e.g., alpha peak and line noise) the cross-spectral slope can be obtained almost with equivalent precision from the mixed (raw) cross-power spectrum. The relevance of MRCSA, however, is well demonstrated in estimating the percentage of fractal cross-spectral power under these conditions (right panel). It is evident that the presence of even one oscillation can drastically reduce the proportion of fractal cross-spectral power – the highest value is around 80%, while in pure mixed-correlated ARFIMA the obtained value is close to the theoretical 100%. It can be concluded therefore that MRCSA is robust against the presence of multiple oscillatory peaks, as well as it is largely unaffected by the amplitude of oscillatory components.

#### Effect of Noise

**FIGURE 4:**
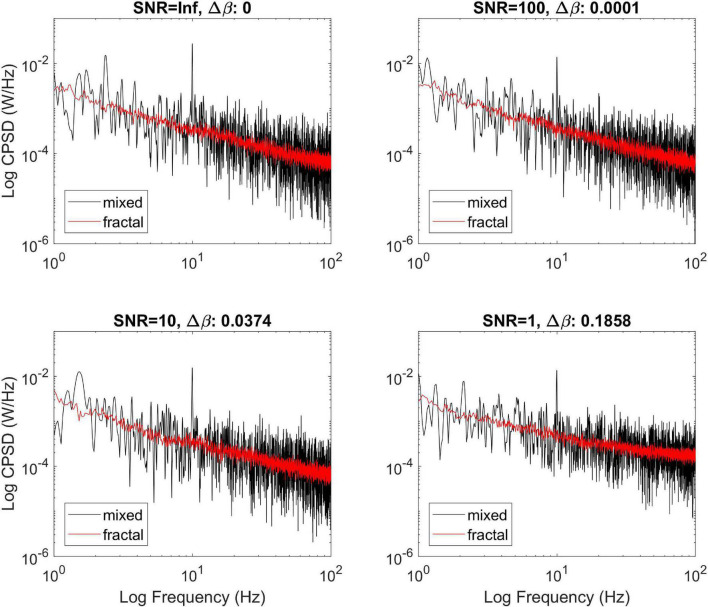
Effect of additive noise on the performance of MRCSA. The effect of noise is illustrated at four different levels of signal to noise ratio. Δ refers to the squared difference between β_*xy*_ estimated from the fractal cross-spectrum before and after adding Gaussian noise.

The effect of additive Gaussian noise is illustrated on Figure 4. All processes were simulated at a true cross-spectral slope of 0.8, with one oscillatory component added at 10 Hz. It appears that white noise introduced a bias to an extent below 5% as long as SNR is high enough (i.e., 10). On the other hand, at low SNR (lower right panel) although MRCSA still readily removed the oscillatory peak, the estimate of the cross-spectral slope became increasingly biased. Nevertheless, MRCSA appeared to be quite robust against the moderate presence of additive noise.

### Real-World Experiment

#### Demonstration

**FIGURE 5:**
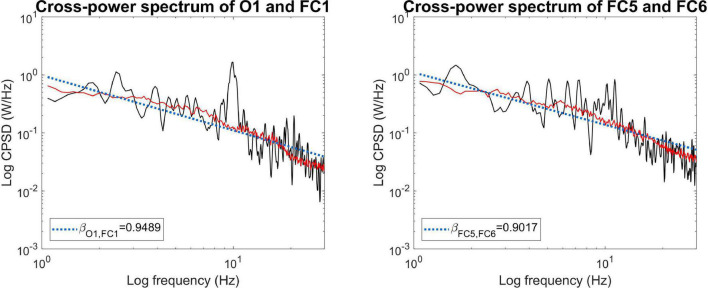
Application of MRCSA on empirical data. MRCSA is effective even when the oscillatory peak spreads to a narrow frequency range **(Left)**. It is also observable that the cross-power spectra of occipito-frontal **(Left)** and **(Right)** fronto-frontal connections can express characteristic differences, such as the apparent lack of increased alpha synchronization in the latter.

First, we show that MRCSA works effectively not only on simulated time series but on real-world experimental data as well. Figure 5 shows the mixed (black) and fractal (red) cross-power spectra in two separate cases. Data came from a representative subject (#4) in WG condition. The prominent alpha peak around 10 Hz can be clearly observed in the mixed cross-power spectrum between regions O1 and FC1, which is efficiently eliminated by MRCSA (left panel). On the other hand, there is much less alpha synchronization observable between regions FC5 and FC6 (right panel). This difference is also captured in the percentage of fractal cross-spectral power, which was obtained as 72.19 and 82.70% for the former and the latter case, respectively.

#### Group-Level Analysis

**FIGURE 6:**
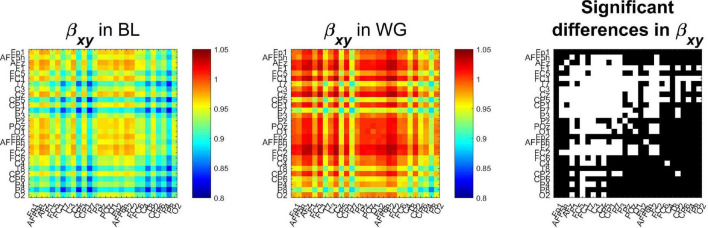
Cross-spectral exponents of functional connections. It can be seen that compared to the baseline condition **(Left)**, during word generation cross-spectral slope increased in most connections **(Middle)**. Those cases where this difference was identified as significant (following Bonferroni adjustment) are marked in white on the **(Right)**. BL, baseline; WG, word generation.

Group-averaged spectral exponents are shown on Figure 6, where the left and middle panels show auto- and cross-spectral exponents from BL and WG, respectively, while the right panel marks those locations in white where the values were significantly different (*p* < 0.05 after Bonferroni adjustment) in the two conditions. Auto-spectral exponents are found in the main diagonals, while indices of the rest of the cells denote the corresponding channel-pairs for the cross-spectral exponents. It is clearly observable that both auto- and cross-spectral slopes increased due to word generation when compared to resting condition. A strong lateralization can also be observed, as functional connections among left hemispherical regions tend to have not only higher exponents in both conditions, but also more connections where the spectral slope increased. In total, 143 of 378 connections were found significantly different in the two conditions. Node degrees were also computed to reduce dimensionality of the data. Indeed, we found that all cortical regions expressed significantly higher node degree in WG than in BL condition (*p* < 0.05 in all cases after Bonferroni adjustment).

**FIGURE 7:**
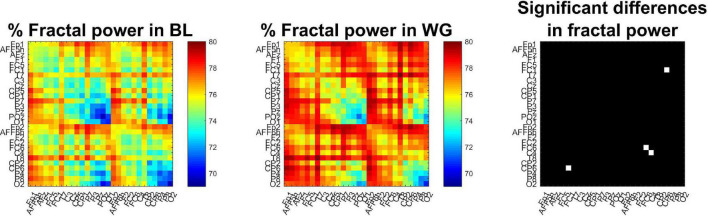
Proportion of fractal power of functional connections. The percentage of fractal cross-spectral power appears generally smaller in baseline **(Left)** when compared to word generation **(Middle)** conditions. The **(Right)** panel marks those channels in white where this increase was significant. Only the connection between regions FC1 and CP6 showed significant difference after Bonferroni adjustment, as well as the percentage of fractal auto-spectral power was higher at regions FC6 and C4, as indicated by the two white cells in the main diagonal.

Fractal spectral power showed similar trends (Figure 7). The proportion of fractal spectral power showed an increasing trend when transitioning from BL to WG, indirectly indicating a reduction in oscillatory auto- and cross-spectral power. Nevertheless, this difference remained tendential, as the increase in fractal cross-spectral power was significant in only one connection (FC1-CP6, *p* = 0.0329 following Bonferroni adjustment), while over two locations (FC6 and C4, *p* = 0.0478 and *p* = 0.0134, respectively, Bonferroni-adjusted) for auto-spectral power. Node degree analysis indicated that connections of regions C4, CP6, and P4 had higher proportion of fractal cross-spectral power in WG when compared to BL (*p* = 0.0235, *p* = 0.0122, and *p* = 0.0478, respectively, Bonferroni-adjusted).

## Discussion

In this work we presented the MRCSA method to separate the fractal component of the cross-power spectrum of two long-range coupled signals. MRCSA is the bivariate extension of IRASA (Wen and Liu, 2016), and thus it builds on the same principles as its univariate counterpart. From this it inherently follows, that MRCSA will be subject to the same considerations and limitations. A comprehensive, in-depth discussion of these aspects are presented in the original paper of Wen and Liu (2016), and thus here we briefly summarize them along the same lines, before turning to discussing the potential utilities and future perspectives of MRCSA itself.

### Considerations Regarding the Multiple-Resampling Cross-Spectral Analysis Method

#### The Multiple-Resampling Scheme

For the sake of consistency, here we utilized the originally proposed set of rescaling factors, namely *h* was ranging between 1.1 and 1.9 in 0.05 increments. Just like in case of IRASA, when utilizing MRCSA one can select an arbitrary set of reasonable rescaling factors (as long as *h* > 0), as in theory the technique is independent from the actual values of *h*. Nevertheless, one must pay attention to practical considerations: the accessible frequency range is half of the sampling rate of the investigated processes (as according to the Nyquist sampling theorem) and resampling with a factor of *2* further decreases this to one quarter. Therefore, selecting a wider range of resampling factors might provide a better reconstruction of the fractal (cross-)spectrum at the cost of reducing the accessible range of frequencies (Wen and Liu, 2016). This is true in the opposite direction as well: reducing the range of *h* might allow for a broader reconstruction of the fractal spectrum. However, in this case one has to pay attention to another phenomenon when applying IRASA/MRCSA. In case of neurophysiological signals, it is common that oscillatory components do not only appear at a single frequency but instead rather appear as a ‘bump’ spreading across a narrow frequency range, such as the alpha peak that is typically localized in the 9–12 Hz (Buzsaki et al., 2012). In this case using a too small range of *h* values (such as *h*ε[1.05;1.5] in 0.05 increments) would only ‘smear’ the peak into two smaller bumps below and over the central frequency of the narrow-band component (Racz et al., 2021). This is a direct result of the central principle behind IRASA and MRCSA: although the alpha peak gets relocated with each down- and upsampling, since the resampling factors are not large enough the outside parts of the relocated peaks overlap and thus result in remnant oscillatory peaks (Racz et al., 2021). Note that in case of overlap at the largest *h* value this effect cannot be compensated by increasing the number of rescaling factors within the given range if the overlap, as the number of outliers at the affected frequencies will never drop below 50%. Therefore, one always has to consider the sampling frequency and the desired frequency range of interest when setting the values of *h*. Given that in most cases EEG data is being sampled at ∼500 Hz (or over) and the frequency range of interest is below 100 Hz, the initial settings of *h*ε[1.1;1.9] appeared as a reasonable and pragmatic choice. Similar considerations were made when analyzing the EEG data in this study: since we preferred the elimination of narrow-band peaks in the cross-spectrum (as seen in Figure 5), we decided to use this range of *h* values and in turn focus only on the frequency range below 30 Hz.

#### Multiple Scaling Ranges

Irregular-resampling auto-spectral analysis was also evaluated by Wen and Liu (2016) in scenarios where the auto-power spectrum of the signal had more than one scaling ranges with different spectral slopes. This phenomenon is indeed relevant as multimodality of the auto-power spectrum is often observed in a variety of physiological processes (Eke et al., 2006; He et al., 2010; Nagy et al., 2017; Mukli et al., 2018; Racz et al., 2021). Although in this work we did not consider such scenarios, given that IRASA and MRCSA work along the same principles, similar considerations can be made for plausible future extensions in this direction. Even though multiple-resampling by non-integer factors does not affect the location of the breakpoint in the fractal spectrum, it does have a ‘blurring’ effect (Wen and Liu, 2016). Notably, this blurring effect also depends on the range of resampling factors: a wider range of *h* might provide a better elimination of narrow-band peaks, however, it will also result in more substantial smoothing of sharp breakpoints (Racz et al., 2021).

#### Advantage of Multiple-Resampling Cross-Spectral Analysis Over Native Cross-Spectral Analysis

From our results presented on Figure 3 it is also apparent, that in cases when the contribution of the oscillatory components is not substantial, MRCSA yields only marginally better estimates of the cross-spectral slope than analyzing the raw (mixed) cross-spectrum itself. This result is not necessarily surprising, as similar observations were made when the CGSA method was introduced (Yamamoto and Hughson, 1993). On the other hand, the percentage of fractal cross-spectral power was substantially affected (∼20% drop) even with one oscillatory component with small amplitude present; an effect which could only be captured by applying MRCSA. This gives MRCSA another utility apart from obtaining the cross-spectral slope from an unbiased estimate of the cross-power spectrum, which might carry relevance in physiological time series analysis. In light of this the results obtained from real-world EEG data are somewhat disappointing, as the percentage of fractal cross-spectral power showed a tendency of increasing broadly over the cortex, although this difference was only identified as significant in case of a single connection (see Figure 7) after applying Bonferroni correction. Given that the use of Bonferroni correction is often questioned in natural sciences (Perneger, 1998) we re-evaluated the obtained results for exploratory reasons with using a less conservative technique for multiple comparisons adjustment, the false discovery rate (FDR) method of Benjamini and Hochberg (1995) with a level of significance *p* < 0.05. This analysis reflected more closely the patterns observed on Figure 7, showing that the proportion of fractal cross-spectral power was indeed higher in the WG condition in 143 of the 378 connections when compared to BL (see Supplementary Figure 1). Note that applying this correction procedure (instead of Bonferroni adjustment) to the obtained cross-spectral exponents indicated a significantly larger slope in WG for all connections.

### Simulation Environment

Here we utilized the framework of mixed-correlated ARFIMA processes (Kristoufek, 2013a) for generating time series pairs with known bivariate Hurst exponents/cross-spectral slopes. The mixed-correlated ARFIMA method provides probably the most sophisticated and versatile framework yet for simulating bivariate long-range cross-correlated processes, however, some considerations have to be made at its application. Notably, ARFIMA processes are integrated from *n=0* to ∞ in the past [see Eqs. (23) and (24)]. In case of simulated data this leads to unavoidable finite size effects, as well as the weights *a*_*n*_(*d*) can only be reliably computed with relatively small *n* (e.g., *n* < 100) before numerical instabilities become intolerable. Therefore, it can be anticipated that the univariate and bivariate Hurst-exponents could only be estimated from simulated mixed-correlated ARFIMA time series with some unavoidable error. Indeed, it was found in multiple simulation settings that irrespective of the estimator – let it be in the time or in the frequency domain –, Hurst exponents obtained from simulated time series deviated from their expected true values; a bias that was also dependent on the parametrization of the mixed-correlated ARFIMA model as well as the estimators themselves (Kristoufek, 2014, 2017). Given that our goal here was to demonstrate the ability of MRCSA to remove oscillatory peaks from the cross-power spectrum, we decided to (i) focus only on the bivariate spectral slopes β_*xy*_, and (ii) compare the obtained β_*xy*_ values not to the expected theoretical values but rather to those estimated from the naive simulated mixed-correlated ARFIMA time series (i.e., before adding sinusoidal components and/or Gaussian noise). This testing procedure better reflected the true effect of MRCSA itself without possible bias that might be introduced by the procedure used for generating simulated data.

Although in the *in silico* testing time series are generated with known fractal characteristics satisfying the power-law relationship, in case of empirical data the presence of the power-law relationship first needs to be validated by an appropriate statistical framework (Clauset et al., 2009) as a precondition to render the subsequent fractal analysis meaningful. Here we proposed a frequency domain method for providing an unbiased estimate for scale-free component based on cross-spectral power densities. Given that by extending the Weiner–Khintchine theorem the cross-power spectrum appears as the Fourier transform of the cross-correlation function (Prichard and Theiler, 1994), our proposed MRCSA algorithm captures linear characteristics of the data. However, it is of further interest whether the identified scale-free component of the cross-spectrum can truly be attributed to the long-term memory of the bivariate processes or it should be regarded as statistical noise due to heavy-tailed distribution of the data (Kantelhardt et al., 2002; Wu et al., 2018). Generally, such cases can (and should) be distinguished by refinement of shuffling tests introduced earlier for fractal time series analysis (Racz et al., 2019, 2020; Stylianou et al., 2020). Nevertheless, given that the main purpose of analyzing empirical data in this work was to demonstrate the efficacy of MRCSA, for the sake of simplicity this step was omitted.

### Bivariate Fractal and Scale-Free Dynamics

It is also important to make a distinction between ‘fractal’ and ‘scale-free’ characteristic, even though the two terms are often used synonymously. An arbitrary process (such as linearly filtered noise) might produce a power spectrum that obeys a *F*(ω)αω^β^ relationship without carrying any practical relevance, while it has been shown that one of the characteristics of fractal processes is a random phase distribution in the complete range of [0;π] (Yamamoto and Hughson, 1993). The cross-spectrum on the other hand does not depend on the phases of the individual time series, but instead on the differences of the phase angles (Prichard and Theiler, 1994). Given that MRCSA extracts the fractal component of the cross-power spectrum it does not contain phase information. One implication of this is that – similarly to IRASA – the extracted components may or may not reflect actual physiological significance and thus one always has to apply caution when interpreting such results. Secondly, MRCSA by itself characterizes only linear coupling between processes (Prichard and Theiler, 1994), while for assessing non-linear interdependence one has to turn to different methods such as chaotic time series analysis (Bezruchko et al., 2008), or approaches based on information theory (Besserve et al., 2010).

Further surrogate testing might also allow for elucidating the origin of the observed bivariate fractality. As previously mentioned in relation to Eq. (16), many previous studies confirmed both theoretically and numerically that the bivariate Hurst exponent (and equivalently the cross-spectral slope) derives as the average of the individual univariate Hurst exponents of the involved processes (Podobnik and Stanley, 2008; Podobnik et al., 2011). Nevertheless, analysis of empirical data often indicates that this is not always true by default; indeed, it can be derived analytically that the bivariate Hurst exponent can be either equal or smaller, but not larger than the average of univariate Hurst exponents (Kristoufek, 2015). In line with this notion, multiple processes have been proposed with such properties (Sela and Hurvich, 2012; Kristoufek, 2013a). Along these lines, in an earlier study we presented a novel method for analyzing bivariate (multi)fractality, with statistical tests for assessment of genuine scale-free coupled dynamics included (Stylianou et al., 2020). In that and a following paper (Stylianou et al., 2021) we introduced the notion of extrinsic and intrinsic bivariate fractality, referring to the cases when *H*_*xy*_ is equal or smaller than the average of *H_x_* and *H_y_*, respectively. In contrast to extrinsic fractality, the presence of intrinsic fractality implies that the properties of the bivariate processes cannot only be explained from those of the univariate processes. In other words, the latter case implicates genuine scale-free coupled processes due to intrinsic interaction of their dynamics, which may not result from pure autocorrelation effects originating from external source. To distinguish between these two cases of bivariate fractality, uni- and bivariate Hurst exponents of the analyzed signal pairs could be compared, which bears significance in fractal connectivity analysis. Although our mixed-correlated ARFIMA-based testing framework would theoretically allow for extending our investigations into these directions, for the sake of clarity and simplicity we omitted this aspect for now. Nevertheless, assessing this aspect of the analyzed data provides means for surrogate thresholding of connections depending on the strength and quality of the scale-free coupling, which is a fundamental question in functional connectivity studies. Thus, future analytical frameworks implementing MRCSA would benefit from surrogate testing procedures for the origin and nature of coupled dynamics, which is beyond the scope of this paper.

### Results From Experimental Electroencephalography Data

By analyzing experimental EEG obtained from a baseline and word generation paradigm we demonstrated that fractal cross-spectral slope can be utilized to distinguish between different mental states. We observed a strong increase in cross-spectral slope in increased mental activity when compared to an idle state. This is in line with previous results identifying an increase in the bivariate Hurst exponent due to performing a cognitive task (Stylianou et al., 2021). The observed results imply that long-range cooperation among distinct brain regions strengthened while performing the word generation task. It is important to stress that an increase in the cross-spectral slope does not necessarily mean increased/strengthened synchronization, but instead it reflects that coupling between the two processes remains significant even on ever longer time scales (Kristoufek, 2015). This might be understood based on the nature of the task: the word generation paradigm involved increased association between many higher order brain functions, such as short- and long-term memory, association and attention. Furthermore, the subjects had to sustain this increased cooperation for 10 s, which might have indeed resulted in increased long-term coupling between the involved brain regions. In line with this hypothesis, most increase in β_*xy*_ could be observed in connections linking frontal and prefrontal regions and parietal regions (see Figure 6), cortical areas that can be mostly associated with dorsal and ventral attention networks (Yeo et al., 2011; Racz et al., 2019). It has to be noted, however, that conclusions regarding the underlying cortical regions cannot be made with certainty based solely on source-space EEG data (Giacometti et al., 2014). Simultaneously to the increase in cross-spectral slope, our analysis revealed an increasing tendency in the proportion of fractal cross-spectral power of functional connections. These results indirectly suggest a decrease in narrow-band synchronization, which might reflect the alpha desynchronization commonly observed during intense mental workload (Klimesch et al., 1997).

Despite intense research, the physiological role and significance of fractal brain activity is yet not completely understood and thus it is a subject of long-lasting debate in the scientific community. Substantial skepticism stems from the fact that fractal dynamics are ubiquitous in many natural processes (Bak, 1996; Gisiger, 2001), while other approaches simply consider fractal neural dynamics as ‘1/*f* noise’ (Mitra and Pesaran, 1999). On the other hand it has been observed in many studies that scale-free characteristics of brain dynamics change in relation to physiological state such as increased mental workload (He et al., 2010; He, 2011; Ciuciu et al., 2012; Zilber et al., 2012), anxiety (Tolkunov et al., 2010) or self-consciousness (Huang et al., 2016; Kolvoort et al., 2020). Nevertheless, the vast majority of previous studies considered univariate fractal dynamics and not long-term correlations in interregional connections. On the other hand, only a handful of studies analyzed fractal brain connectivity (Achard et al., 2008; Wang and Zhao, 2012; Stylianou et al., 2020; La Rocca et al., 2021) and even less assessed its changes due to performing a cognitive task (Ciuciu et al., 2014; Stylianou et al., 2021). Our results contribute to the growing body of literature suggesting that fractal brain connectivity has functional significance as it can differentiate between various mental states, in which capacity MRCSA could prove as a valuable tool in future studies.

### Future Perspectives and Plausible Developments

Although we demonstrated that MRCSA is effective in separating the fractal component of the cross-power spectrum, a number of considerations and assumptions must be made at its application, as touched upon previously. Nevertheless, theoretical and technical development in the future might offer solutions for or extend upon some of the shortcomings of the currently presented method. The extension of MRCSA to the bimodal domain might be pursued along the lines of Nagy et al. (2017) and Mukli et al. (2018). Additionally, in many natural fractal processes the scaling property itself can vary over time, meaning that dynamics of such processes cannot be fully characterized by a single scaling exponent. This can be achieved instead by estimating a set of scaling exponents; a phenomenon called *multifractality* (Benzi et al., 1984; Mandelbrot, 1986). Multifractality has been confirmed in many physiological processes such as heart rate variability (Ivanov et al., 1999) or cerebral hemodynamics (Shimizu et al., 2004). Importantly, functional brain connectivity has also been shown recently to express not only fractal but indeed multifractal dynamics on the level of global network topological properties (Racz et al., 2018a,^2020^) as well as individual connections (Racz et al., 2018b; Stylianou et al., 2020, 2021). The presented MRCSA algorithm by itself is only capable of characterizing the global *monofractal* character of functional coupling, however, by utilizing a sliding window approach one can actually obtain a trajectory/distribution of local cross-spectral exponents over time. Then, the distribution width can be used to characterize the degree of multifractality, while the center of distribution describes the global monofractal scaling. Finally, MRCSA could be utilized in disciplines other than neuroscience, as well as complementary to other analysis techniques. In fact, assessing long-range coupling between processes so far gained the most attention in financial data analysis (Podobnik and Stanley, 2008; Podobnik et al., 2009; He and Chen, 2011; Pal et al., 2014), but applications are also found in the fields of geophysics (Marinho et al., 2013), molecular biology (Stan et al., 2013) or traffic flow data analysis (Xu et al., 2010). Financial time series in particular often express long-range auto- and cross-correlations, while at the same time are affected by common periodic trends (e.g., daily/yearly periods and business cycles) introducing enhanced synchronization at specific frequencies (He and Chen, 2011). MRCSA provides a solution for eliminating the confounding effects of multiple periodic trends simultaneously, and thus it can become an important tool for financial data analysis, too. Furthermore, it can also be used to complement other analysis methods from the fields of chaos theory (Abarbanel et al., 1993; Anishchenko, 2007; Glushkov, 2012), non-linear analysis (Takens, 1981; Grassberger and Procaccia, 1983; Khetselius, 2013) or information theory (Schreiber, 2000; Besserve et al., 2010), among others, for a better understanding of complex dynamical systems.

## Conclusion

In this study we introduced the MRCSA algorithm for separating the fractal component of the cross-spectral spectrum of long-range coupled signals. MRCSA is the extension of the previously published univariate IRASA method to the bivariate domain, and as such builds on the same principles. We showed that MRCSA efficiently eliminates narrow-band peaks in the cross-power spectrum introduced by correlated oscillatory components in the signals. MRCSA also appeared immune to increasing the number and/or amplitude of correlated oscillatory components, as well as it proved robust against additive Gaussian noise to a moderate extent. Apart from *in silico* simulations we demonstrated the applicability of MRCSA on empirical EEG data as well and showed that cross-spectral slopes could be used to distinguish between resting-state and increased mental workload conditions. MRCSA also carries potential utility in disciplines other than neuroscience, e.g., financial data analysis, where periodic trends might present a similar challenge as narrow-band oscillations in neurophysiological signals.

## Data Availability Statement

Publicly available datasets were analyzed in this study. This data can be found here: http://doc.ml.tu-berlin.de/simultaneous_EEG_NIRS.

## Ethics Statement

The studies involving human participants were reviewed and approved by Ethics Committee of the Institute of Psychology and Ergonomics, Berlin Institute of Technology. The patients/participants provided their written informed consent to participate in this study.

## Author Contributions

FSR designed the study, developed the method, performed simulations and data analysis, and wrote the initial draft of the manuscript. AC, ZK, and OS contributed to performing the simulations, literature review, and manuscript development. PM and AE contributed to deriving the method and manuscript development. All authors have contributed to, reviewed, and approved the final version of the manuscript.

## Conflict of Interest

The authors declare that the research was conducted in the absence of any commercial or financial relationships that could be construed as a potential conflict of interest.

## Publisher’s Note

All claims expressed in this article are solely those of the authors and do not necessarily represent those of their affiliated organizations, or those of the publisher, the editors and the reviewers. Any product that may be evaluated in this article, or claim that may be made by its manufacturer, is not guaranteed or endorsed by the publisher.
