# Supplementary material for: Multiple-Resampling Cross-Spectral Analysis: An Unbiased Tool for Estimating Fractal Connectivity With an Application to Neurophysiological Signals
Source: Front Physiol. 2022 Mar 7;13:817239. doi: 10.3389/fphys.2022.817239 (PMC8936508; doi:10.3389/fphys.2022.817239)
Supplement: Supplementary file 1 [file Data_Sheet_1.docx]

Supplementary material for Multiple-resampling cross-spectral analysis (MRCSA): an unbiased tool for estimating fractal connectivity

Frigyes Samuel Racz, Akos Czoch, Zalan Kaposzta, Orestis Stylianou, Peter Mukli and Andras Eke

# Obtaining the separated oscillatory component of the cross-power spectrum

It has been shown by Wen and Liu (2016) that an unbiased estimate of the oscillatory component of the auto-power spectrum can be obtained as the confounding term $2F\left( \omega\right)X\left( \omega\right)\cos\left( \alpha\left( \omega\right)-\beta\left( \omega\right) \right)$ can be eliminated via averaging from multiple data segments assuming random phase relationship between the fractal and oscillatory components. For the sake of clarity, let’s re-state the formula for the auto-power spectrum of a signal composed of a fractal and oscillatory components:

| $Y^{2}\left( \omega\right)=Y\left( \omega\right)\bar{Y\left( \omega\right)}=F^{2}\left( \omega\right)+X^{2}\left( \omega\right)+2F\left( \omega\right)X\left( \omega\right)\cos\left( \alpha\left( \omega\right)-\beta\left( \omega\right) \right).$ | (S1) |
| --- | --- |

Since the last term on the right-side of the equation has an expected value of 0, it can be eliminated by averaging, and thus assuming stationarity one can obtain

| $\hat{Y^{2}}\left( \omega\right)=Y\left( \omega\right)\bar{Y\left( \omega\right)}=F^{2}\left( \omega\right)+X^{2}\left( \omega\right),$ | (S2) |
| --- | --- |

where $\hat{Y^{2}}\left( \omega\right)$ denotes the averaged squared auto-spectral power at frequency $\omega$ obtained by averaging the auto-spectral power over multiple data segments. Now, let’s derive the squared cross-power spectrum ${SS}_{xy}^{2}(\omega)$ of two composite signals $x\left( t \right)=f_{x}\left( t \right)+h_{x}(t)$ and $y\left( t \right)=f_{y}\left( t \right)+h_{y}(t)$, each consisting of a fractal and an oscillatory component. Applying the same formula

| ${SS}_{xy}^{2}\left( \omega\right)=\left[ X\left( \omega\right)\bar{Y\left( \omega\right)} \right]^{2}=\left[ X\left( \omega\right)\bar{Y\left( \omega\right)} \right]\bar{\left[ X\left( \omega\right)\bar{Y\left( \omega\right)} \right].}$ | (S3) |
| --- | --- |

In more detail, Eq. (S3) yields

| ${SS}_{xy}^{2}\left( \omega\right)=\left[ FX\left( \omega\right)e^{-j\alpha\left( \omega\right)}+HX\left( \omega\right)e^{-j\beta\left( \omega\right)} \right]\times\left[ FY\left( \omega\right)e^{j\gamma\left( \omega\right)}+HY\left( \omega\right)e^{j\delta\left( \omega\right)} \right]\times\left[ FX\left( \omega\right)e^{j\alpha\left( \omega\right)}+HX\left( \omega\right)e^{j\beta\left( \omega\right)} \right]\times\left[ FY\left( \omega\right)e^{-j\gamma\left( \omega\right)}+HY\left( \omega\right)e^{-j\delta\left( \omega\right)} \right].$ | (S4) |
| --- | --- |

After excluding all terms one arrives to the following equation

| ${SS}_{xy}^{2}\left( \omega\right)={FX}^{2}\left( \omega\right){FY}^{2}\left( \omega\right)+{FX}^{2}\left( \omega\right){HY}^{2}\left( \omega\right)+{FY}^{2}\left( \omega\right){HX}^{2}\left( \omega\right)+{HX}^{2}\left( \omega\right){HY}^{2}\left( \omega\right)+A\left( \omega\right)+B\left( \omega\right)+C\left( \omega\right)+D\left( \omega\right)+E\left( \omega\right)+F\left( \omega\right),$ | (S5) |
| --- | --- |

where the terms are the following:

| $A\left( \omega\right)=2{FX}^{2}\left( \omega\right)FY\left( \omega\right)HY\left( \omega\right)\cos\left( \gamma\left( \omega\right)-\delta\left( \omega\right) \right),$  $B\left( \omega\right)=2{FY}^{2}\left( \omega\right)FX\left( \omega\right)HX\left( \omega\right)\cos\left( \alpha\left( \omega\right)-\beta\left( \omega\right) \right),$  $C\left( \omega\right)=2FX\left( \omega\right){HX}^{2}\left( \omega\right)HX\left( \omega\right)\cos\left( \alpha\left( \omega\right)-\beta\left( \omega\right) \right),$  $D\left( \omega\right)=2FY\left( \omega\right){HX}^{2}\left( \omega\right)HY\left( \omega\right)\cos\left( \gamma\left( \omega\right)-\delta\left( \omega\right) \right),$  $E\left( \omega\right)=2FX\left( \omega\right)FY\left( \omega\right)HX\left( \omega\right)HY\left( \omega\right)\cos\left( \left( \beta\left( \omega\right)-\gamma\left( \omega\right) \right)-\left( \alpha\left( \omega\right)-\delta\left( \omega\right) \right) \right)$ and  $F\left( \omega\right)=2FX\left( \omega\right)FY\left( \omega\right)HX\left( \omega\right)HY\left( \omega\right)\cos\left( \left( \beta\left( \omega\right)-\delta\left( \omega\right) \right)-\left( \alpha\left( \omega\right)-\gamma\left( \omega\right) \right) \right).$ |  |
| --- | --- |

It can be seen that when assuming a random relationship between the phases of *all* fractal and oscillatory components, the terms $A\left( \omega\right)$ to $F\left( \omega\right)$ have an expectation of zero, and thus can be eliminated by averaging over multiple time segments. However, the terms ${FX}^{2}\left( \omega\right){HY}^{2}\left( \omega\right)$ and ${FY}^{2}\left( \omega\right){HX}^{2}\left( \omega\right)$ do not depend on the phase and thus unaffected by averaging, which is the reason why the oscillatory component of the cross-power spectrum cannot simply be obtained by subtracting the fractal component. Nevertheless, if one computes the auto-power spectrum of $x\left( t \right)$ and $y\left( t \right)$ by means of IRASA and obtains

| $\hat{X^{2}}\left( \omega\right)={FX}^{2}\left( \omega\right)+{HX}^{2}\left( \omega\right)$ and  $\hat{Y^{2}}\left( \omega\right)={FY}^{2}\left( \omega\right)+{HY}^{2}\left( \omega\right)$, | (S6) |
| --- | --- |

then these terms can also be reconstructed, and thus a separate estimate on the oscillatory component can also be obtained.

# Fractal spectral power

When comparing proportion of the fractal cross-spectral power in the baseline and word generation states, only one of the connections revealed a significant increase following Bonferroni adjustment. Nevertheless, when applying a less conservative adjustment technique, namely the false discovery rate method of Benjamini and Hochberg (1995), we found that 143 of the 378 connections expressed a significantly higher proportion of cross-spectral power in the word generation when compared to the baseline condition ($p<0.05$ following Benjamini-Hochberg adjustment). These results are illustrated on **Supplementary Figure S1**.

**
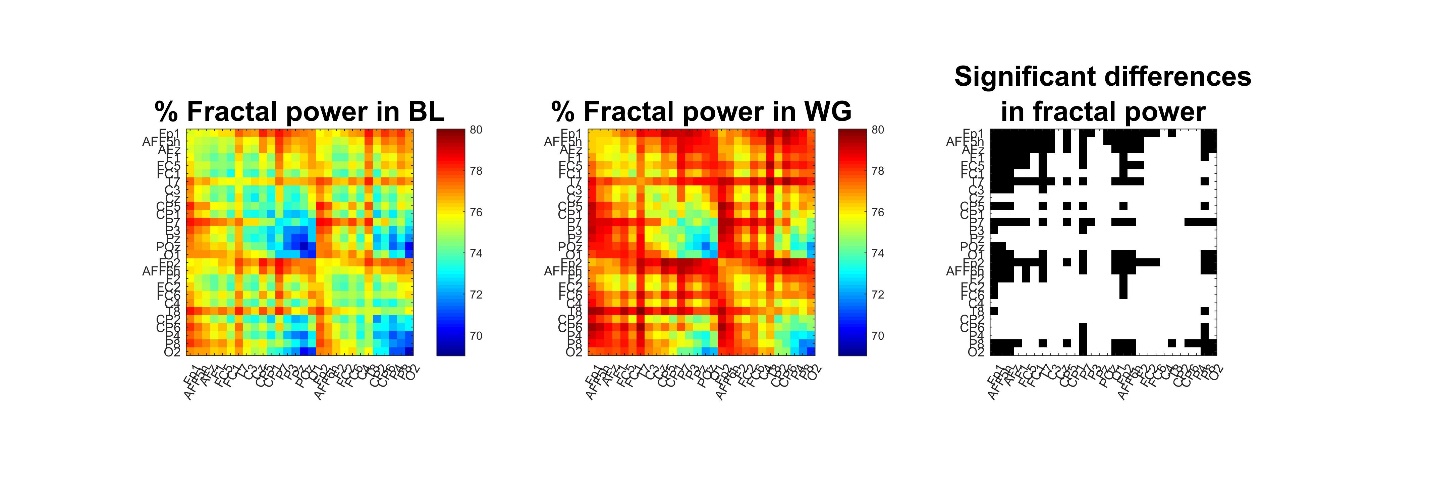
**

**Supplementary Figure S1.** Proportion of fractal power of functional connections. The percentage of fractal cross-spectral power appears generally smaller in baseline (left) when compared to word generation (middle) conditions. The right panel marks those channels in white where this increase was significant. 143 out of 378 connections showed a significant increase following Benjamini-Hochberg adjustment.

**References**

BENJAMINI, Y. & HOCHBERG, Y. 1995. Controlling the False Discovery Rate - a Practical and Powerful Approach to Multiple Testing. *Journal of the Royal Statistical Society Series B-Statistical Methodology,* 57**,** 289-300.

WEN, H. G. & LIU, Z. M. 2016. Separating Fractal and Oscillatory Components in the Power Spectrum of Neurophysiological Signal. *Brain Topography,* 29**,** 13-26.
