# Supplementary figures and images for: Multiple-Resampling Cross-Spectral Analysis: An Unbiased Tool for Estimating Fractal Connectivity With an Application to Neurophysiological Signals
Source: Front Physiol. 2022 Mar 7;13:817239. doi: 10.3389/fphys.2022.817239 (PMC8936508; doi:10.3389/fphys.2022.817239)

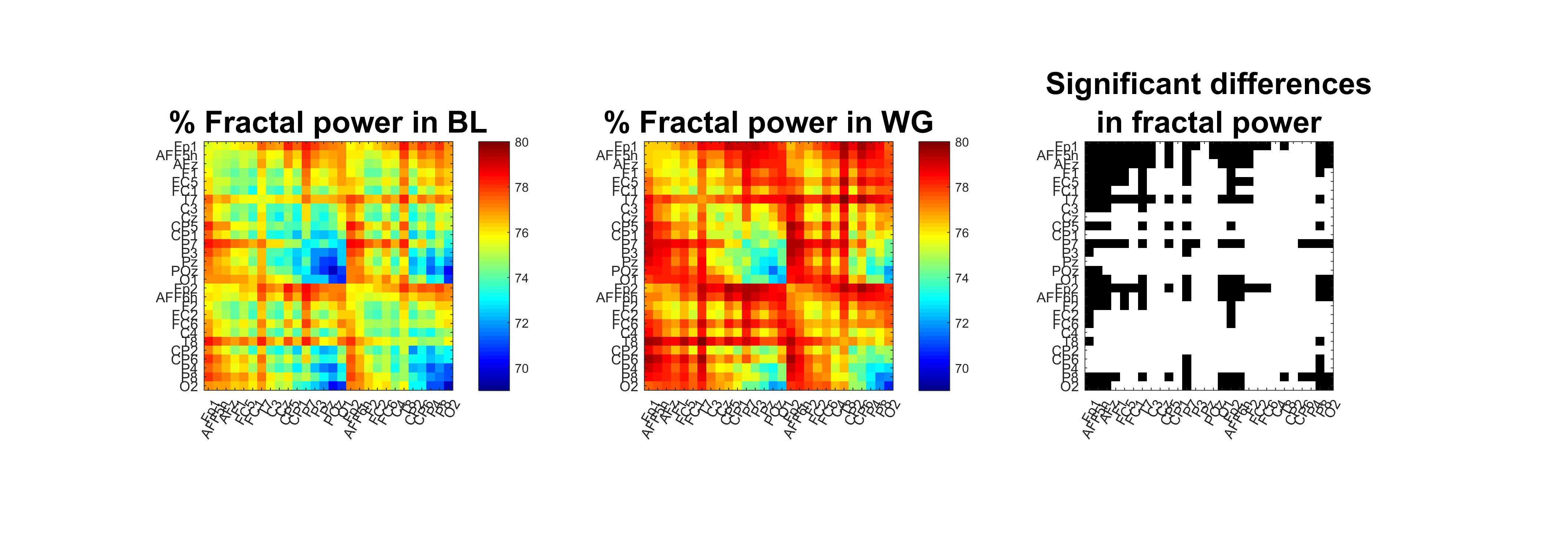

Supplement: Supplementary file 2 [file Image_1.JPEG]
